# Supplementary material for: Development and Validation of Reverse Transcriptase Loop-Mediated Isothermal Amplification (RT-LAMP) as a Simple and Rapid Diagnostic Tool for SARS-CoV-2 Detection
Source: Diagnostics (Basel). 2022 Sep 15;12(9):2232. doi: 10.3390/diagnostics12092232 (PMC9498013; doi:10.3390/diagnostics12092232)
Supplement: Supplementary file 1 [file diagnostics-12-02232-s001.zip › diagnostics-1823942-supplementary.pdf]

Supplementary Material Section

S1. Designing of Primers

Six specific LAMP primers were designed to target a small fragment of *ORF1a, N* genes and RNAse P as demonstrated in Tables S1, S2 and S3, respectively, using an online primer Explorer V5 (https://primerexplorer.jp).

Table S1. LAMP primer design for *ORF1a* gene.

1	Label	Sequence
	ORF1a _A_F3	**GAAAAGAAAAAGCTTGATGGC**
	ORF1a _A_B3	**GGTAAGTAACCACAAGTAGTG**
	ORF1a _A_FIP	**GAGAGTTGAAAGGCACATTTGGTTG-TTTATGGGTAGAATTCGATCTG**
	ORF1a _A_BIP	**CAGACGGGCGATTTTGTTAAAGC-CACCTTCTTTAGTCAAATTCTCA**
	ORF1a _A_F2	**TTTATGGGTAGAATTCGATCTG**
	ORF1a _A_F1c	**GAGAGTTGAAAGGCACATTTGGTTG**
	ORF1a _A_B2	**CACCTTCTTTAGTCAAATTCTCA**
	ORF1a _A_B1c	**CAGACGGGCGATTTTGTTAAAGC**
	ORF1a _A_LF	**TGGTGACGCAACTGGATAGA**
	ORF1a _A_LB	**CACTTGCGAATTTTGTGGCACTG**
**2**	**Label**	**Sequence**
	ORF1a _B_F3	**GCTGTTGTTAAAATTTATTGTCCAG**
	ORF1a _B_B3	**TATGTTAGCGCTAGCACG**
	ORF1a _B_FIP	**GGTTTTCAAGCCAGATTCATTATGG-CATGTCACAATTCAGAAGTAGG**
	ORF1a _B_BIP	**GTAAGGGTGGTCGCACTATTG-CCCAATAGGCACACTTGTT**
	ORF1a _B_F2	**CATGTCACAATTCAGAAGTAGG**
	ORF1a _B_F1c	**GGTTTTCAAGCCAGATTCATTATGG**
	ORF1a _B_B2	**CCCAATAGGCACACTTGTT**
	ORF1a _B_B1c	**GTAAGGGTGGTCGCACTATTG**
	ORF1a _B_LF	**CGGCAAGACTATGCTCAGGT**
	ORF1a _B_LB	**CCTTTGGAGGCTGTGTGTTC**
**3**	**Label**	**Sequence**
	ORF1a _C_F3	**TGCGTCACCAAATGAATG**

	ORF1a _C_B3	**TGTGACATGCTGGACAAT**
	ORF1a _C_FIP	**GCCCGTCTGCCATGAAGTTT-CAAATGTGCCTTTCAACTCTC**
	ORF1a _C_BIP	**ACTTGCGAATTTTGTGGCACT-ACAACAGCATTTTGGGGTA**
	ORF1a _C_F2	**CAAATGTGCCTTTCAACTCTC**
	ORF1a _C_F1c	**GCCCGTCTGCCATGAAGTTT**
	ORF1a _C_B2	**ACAACAGCATTTTGGGGTA**
	ORF1a _C_B1c	**ACTTGCGAATTTTGTGGCACT**
	ORF1a _C_LF	**CACCACAATGATCACACTTC**
	ORF1a _C_LB	**TTGACTAAAGAAGGTGCCACTA**
**4**	**Label**	**Sequence**
	ORF1a _D_F3	**AATGCAACCAAATGTGCC**
	ORF1a _D_B3	**TCCTACTTCTGAATTGTGACAT**
	ORF1a _D_FIP	**AAGTGGCTTTAACAAAATCGCCC-TTTCAACTCTCATGAAGTGTGA**
	ORF1a _D_BIP	**TGAGAATTTGACTAAAGAAGGTGCC-GCTGGACAATAAATTTTAACAACAG**
	ORF1a _D_F2	**TTTCAACTCTCATGAAGTGTGA**
	ORF1a _D_F1c	**AAGTGGCTTTAACAAAATCGCCC**
	ORF1a _D_B2	**GCTGGACAATAAATTTTAACAACAG**
	ORF1a _D_B1c	**TGAGAATTTGACTAAAGAAGGTGCC**
	ORF1a _D_LF	**ACTACTTGTGGTTACTTACCCCA**
	ORF1a _D_LB	**GCCATGAAGTTTCACCACAATGA**
**5**	**Label**	**Sequence**
	ORF1a _E_F3	**AGCATAGTCTTGCCGAAT**
	ORF1a _E_B3	**GGTTGTCATTAAGACCTTCG**
	ORF1a _E_FIP	**GCCTCCAAAGGCAATAGTGC-TAATGAATCTGGCTTGAAAACC**
	ORF1a _E_BIP	**TGTTCTCTTATGTTGGTTGCCATA-CACCTGTATGGTTACAACCTA**
	ORF1a _E_F2	**TAATGAATCTGGCTTGAAAACC**
	ORF1a _E_F1c	**GCCTCCAAAGGCAATAGTGC**
	ORF1a _E_B2	**CACCTGTATGGTTACAACCTA**
	ORF1a _E_B1c	**TGTTCTCTTATGTTGGTTGCCATA**
	ORF1a _E_LF	**GACCACCCTTACGAAG**
	ORF1a _E_LB	**ACAAGTGTGCCTATTGG**

**6**	**DNA fragment of ORF1a gene 851- 1361 bp**	**TTCAACCAAGGGTTGAAAAGAAAAAGCTTG ATGGCTTTATGGGTAGAATTCGATCTGTCTA TCCAGTTGCGTCACCAAATGAATGCAACCA AATGTGCCTTTCAACTCTCATGAAGTGTGAT CATTGTGGTGAAACTTCATGGCAGACGGGC GATTTTGTTAAAGCCACT GCGAATTTTGTGGCACTGAGAATT GACTAAAGAAGGTGCCACTACTTGTGGTTA CTTACCCCAAAATGCTGTTGTTAAAATTTAT TGTCCAGCATGTCACAATTCAGAAGTAGGA CCTGAGCATAGTCTTGCCGAATACCATAATG AATCTGGCTTGAAAACCATTCTTCGTAAGGG TGGTCGCACTATTGCCTTTGGAGGCTGTGTG TTCTCTTATGTTGGTTGCCATAACAAGTGTG CCTATTGGGTTCCACGTGCTAGCGCTAACAT AGGTTGTAACCATACAGGTGTTGTTGGAGA AGGTTCCGAAGGTCTTAATGACAACCTTCTT GAAATAC**

Table S2. LAMP primer design for *N* gene.

**1**	**Label**	**Sequence**
	N gene_A_F3	**TGGCTACTACCGAAGAGCT**
	N gene_A_B3	**TGCAGCATTGTTAGCAGGAT**
	N gene_A_FIP	**TCTGGCCCAGTTCCTAGGTAGT-GACGAATTCGTGGTGGTGA**
	N gene_A_BIP	**AGACGGCATCATATGGGTTGCA-GCGGGTGCCAATGTGATC**
	N gene_A_F2	**GACGAATTCGTGGTGGTGA**
	N gene_A_F1c	**TCTGGCCCAGTTCCTAGGTAGT**
	N gene_A_B2	**GCGGGTGCCAATGTGATC**
	Ngene_A_B1c	**AGACGGCATCATATGGGTTGCA**
	N gene_A_LF	**GGACTGAGATCTTTCATTTTACCGT**
	N gene_A_LB	**ACTGAGGGAGCCTTGAATACA**
**2**	**Label**	**Sequence**
	N gene_B_F3	**AGATCACATTGGCACCCG**
	N gene_B_B3	**CCATTGCCAGCCATTCTAGC**
	N gene_B_FIP	**TGCTCCCTTCTGCGTAGAAGC-**

		**CAATGCTGCAATCGTGCTAC**
	N gene_B_BIP	**GGCGGCAGTCAAGCCTCTTC-CCTACTGCTGCCTGGAGTT**
	N gene_B_F2	**CAATGCTGCAATCGTGCTAC**
	N gene_B_F1c	**TGCTCCCTTCTGCGTAGAAGC**
	N gene_B_B2	**CCTACTGCTGCCTGGAGTT**
	N gene_B_B1c	**GGCGGCAGTCAAGCCTCTTC**
	N gene_B_LF	**GCAATGTTGTTCCTTGAGGAAGTT**
	N gene_B_LB	**GTTCCTCATCACGTAGTCGCAACA**
**3**	**Label**	**Sequence**
	N gene_C_F3	**GCCAAAAGGCTTCTACGCA**
	N gene_C_B3	**TTGCTCTCAAGCTGGTTCAA**
	N gene_C_FIP	**TCCCCTACTGCTGCCTGGAG-GCAGTCAAGCCTCTTCTCG**
	N gene_C_BIP	**TCTCCTGCTAGAATGGCTGGCA-TCTGTCAAGCAGCAGCAAAG**
	N gene_C_F2	**GCAGTCAAGCCTCTTCTCG**
	N gene_C_F1c	**TCCCCTACTGCTGCCTGGAG**
	N gene_C_B2	**TCTGTCAAGCAGCAGCAAAG**
	N gene_C_B1c	**TCTCCTGCTAGAATGGCTGGCA**
	N gene_C_LF	**TGTTGCGACTACGTGATGAGGA**
	N gene_C_LB	**ATGGCGGTGATGCTGCTCT**
**4**	**Label**	**Sequence**
	N gene_D_F3	**TGGACCCCAAAATCAGCG**
	N gene_D_B3	**GCCTTGTCCTCGAGGGAAT**
	N gene_D_FIP	**CCACTGCGTTCTCCATTCTGGT-AAATGCACCCCGCATTACG**
	N gene_D_BIP	**CGCGATCAAAACAACGTCGGC-CCTTGCCATGTTGAGTGAGA**
	N gene_D_F2	**AAATGCACCCCGCATTACG**
	N gene_D_F1c	**CCACTGCGTTCTCCATTCTGGT**
	N gene_D_B2	**CCTTGCCATGTTGAGTGAGA**
	N gene_D_B1c	**CGCGATCAAAACAACGTCGGC**
	N gene_D_LF	**TTGAATCTGAGGGTCCACCAAA**
	N gene_D_LB	**GTTTACCCAATAATACTGCGTCTTG**

5	Label	Sequence
	N gene_E_F3	**CCAGAATGGAGAACGCAGTG**
	N gene_E_B3	**CCGTCACCACCACGAATT**
	N gene_E_FIP	**AGCGGTGAACCAAGACGCAG GGCGCGATCAAAACAACG**
	N gene_E_BIP	**AATTCCCTCGAGGACAAGGCG- AGCTCTTCGGTAGTAGCCAA**
	N gene_E_F2	**GGCGCGATCAAAACAACG**
	N gene_E_F1c	**AGCGGTGAACCAAGACGCAG**
	N gene_E_B2	**AGCTCTTCGGTAGTAGCCAA**
	N gene_E_B1c	**AATTCCCTCGAGGACAAGGCG**
	N gene_E_LF	**TTATTGGGTAAACCTTGGGGC**
	N gene_E_LB	**TTCCAATTAACACCAATAGCAGTCC**
**6**	**DNA fragment of N gene 1260bp**	**ATGTCTGATAATGGACCCCAAAATCAGCGAAATGCA C CCCGCATTACGTTTGGTGGACCCTCAGATTCAACTG GC AGTAACCAGAATGGAGAACGCAGTGGGGCGCGATC A AAACAACGTCGGCCCCAAGGTTTACCCAATAATACT G CGTCTTGGTTCACCGCTCTCACTCAACATGGCAAGG AA GACCTTAAATTCCCTCGAGGACAAGGCGTTCCAATT A ACACCAATAGCAGTCCAGATGACCAAATTGGCTACT A CCGAAGAGCTACCAGACGAATTCGTGGTGGTGACGG T AAAATGAAAGATCTCAGTCCAAGATGGTATTTCTAC T ACCTAGGAACTGGGCCAGAAGCTGGACTTCCCTATG G TGCTAACAAAGACGGCATCATATGGGTTGCAACTGA G GGAGCCTTGAATACACCAAAAGATCACATTGGCACC C**

		**GCAATCCTGCTAACAATGCTGCAATCGTGCTACAAC<br>TT<br>CCTCAAGGAACAACATTGCCAAAAGGCTTCTACGCA<br>G<br>AAGGGAGCAGAGGCGGCAGTCAAGCCTCTTCTCGTT<br>C<br>CTCATCACGTAGTCGCAACAGTTCAAGAAATTCAAC<br>TC<br>CAGGCAGCAGTAGGGGAACTTCTCCTGCTAGAATGG<br>C<br>TGGCAATGGCGGTGATGCTGCTCTTGCTTTGCTGCT<br>GC<br>TTGACAGATTGAACCAGCTTGAGAGCAAAATGTCTG<br>G<br>TAAAGGCCAACAACAACAAGGCCAAACTGTCACTAA<br>G<br>AAATCTGCTGCTGAGGCTTCTAAGAAGCCTCGGCAA<br>A<br>AACGTACTGCCACTAAAGCATACAATGTAACACAAG<br>C<br>TTTCGGCAGACGTGGTCCAGAACAAACCCAAGGAAA<br>T<br>TTTGGGGACCAGGAACTAATCAGACAAGGAACTGAT<br>T<br>ACAAACATTGGCCGCAAATTGCACAATTTGCCCCCA<br>G<br>CGCTTCAGCGTTCTTCGGAATGTCGCGCATTGGCAT<br>GG<br>AAGTCACACCTTCGGGAACGTGGTTGACCTACACAG<br>G<br>TGCCATCAAATTGGATGACAAAGATCCAAATTTCAA<br>A<br>GATCAAGTCATTTTGCTGAATAAGCATATTGACGCA<br>TA<br>CAAAACATTCCCACCAACAGAGCCTAAAAAGGACAA<br>A<br>AAGAAGAAGGCTGATGAAACTCAAGCCTTACCGCAG<br>A**

		**GACAGAAGAAACAGCAAACTGTGACTCTTCTTCCTG C TGCAGATTTGGATGATTTCTCCAAACAATTGCAACA AT CCATGAGCAGTGCTGACTCAACTCAGGCCTAA**

Table S3. LAMP primer design for RNAse P.

	Label	Sequence
	RNase P_F3	**TTGATGAGCTGGAGCCA**
	RNase P_B3	**CACCCTCAATGCAGAGTC**
	RNase P_FIP	**GTGTGACCCTGAAGACTCGGTTTTAGCCACTGACT CGG ATC**
	RNase P_BIP	**CCTCCGTGATATGGCTCTTCGTTTTTTTCTTACATG GCT CTGGTC**
	RNase P_LF	**ATGTGGATGGCTGAGTTGTT**
	**RNase P_LB**	**CATGCTGAGTACTGGACCTC**
